# Living with constant leaking of urine and odour: thematic analysis of socio-cultural experiences of women affected by obstetric fistula in rural Tanzania

**DOI:** 10.1186/s12905-015-0267-1

**Published:** 2015-11-24

**Authors:** Lilian T. Mselle, Thecla W. Kohi

**Affiliations:** Department of Clinical Nursing, Muhimbili University of Health and Allied Sciences, PO Box 65004 Dar es Salaam, Tanzania; Department of Nursing Management, Muhimbili University of Health and Allied Sciences, PO Box 65004, Dar es Salaam, Tanzania

**Keywords:** Obstetric fistula, Social-cultural experiences, Lived experience, Fistula, Tanzania

## Abstract

**Background:**

Obstetric fistula is a worldwide problem that affects women and girls mostly in Sub Saharan Africa. It is a devastating medical condition consisting of an abnormal opening between the vagina and the bladder or rectum, resulting from unrelieved obstructed labour. Obstetric fistula has devastating social, economic and psychological effect on the health and wellbeing of the women living with it. This study aimed at exploring social-cultural experiences of women living with obstetric fistula in rural Tanzania.

**Methods:**

Women living with obstetric fistula were identified from the fistula ward at CCBRT hospital. Sixteen individual semi structured interviews and two (2) focus group discussions were conducted among consenting women. Interviews were transcribed verbatim and transcripts analysed independently by two researchers using a thematic analysis approach. Themes related to the experiences of living with obstetric fistula were identified.

**Results:**

Four themes illustrating the socio-cultural experiences of women living with obstetric fistula emerged from the analysis of women experiences of living with incontinence and odour. These were keeping clean and neat, earning an income, maintaining marriage, and keeping association. Women experiences of living with fistula were largely influenced by perceptions of people around them basing on their cultural understanding of a woman.

**Conclusion:**

Living with fistula reveals women’s day-to-day experiences of social discrimination and loss of control due to incontinence and odour. They cannot work and contribute to the family income, cannot satisfy their husband’s sexual needs and or bear children, and cannot interact with members of the community in social activities. Women experience of living with fistula was influenced by perceptions of people around them. In the eyes of these people, women who leak urine were of less value since they were not capable of carrying out ascribed social roles.

## Background

The levels of maternal mortality and morbidity in Tanzania are among the highest in the world. In the year 2010, more than 450 maternal deaths were reported per 100,000 live births [1] and women who survive complications associated with delivery suffer from long term disability [2]. The most prominent among these complications is obstetric fistula. Obstetric fistula is a hole between a woman's birth passage and one or more of her internal organs, usually the bladder or the rectum. It usually develops after many days of obstructed labour. Obstetric fistula affects more than 2 million women worldwide, the majority of which are in Africa and Asia [3–5]. In Africa, between 30,000 and 130 000 new cases of obstetric fistula develop each year. Due to women’s low social status, poverty, and long distances to the adequate health facility providing emergency obstetric care (EmOC), many women in Tanzania have less access to adequate birth care. Over 2500 new cases of fistula are estimated to occur each year in Tanzania [6]. Overall, obstetric fistula is a results of lack of prompt medical attention, which leads to prolonged, obstructed labour [7]. If the mother survives labour, during the days and weeks after birth, the dead tissue falls away, leaving holes (fistulas), which allow a constant, uncontrollable loss of urine (vesico-vaginal fistula) or stool (recto-vaginal fistula) into the vagina. This trauma is often compounded by the psychological trauma of delivering a stillborn baby. Up to 83 % of obstetric fistula cases, the baby is stillborn or dies within weeks after delivery [8].

Studies on women affected by fistula in resource poor countries found that socio-cultural and health systems factors contribute to occurrences of obstetric fistula. The socio-cultural practices and cultural beliefs play a vital role in prohibiting women from seeking adequate birth care swiftly. Women’s lack of decision-making power, illiteracy, low economic status, gender inequality, malnutrition, and lack of emergency obstetric care [9–15] contributes to the occurrences of the condition. For example, decisions about mobility of women and expenditure on health care are commonly controlled by men. Unless affected women get fistula treatment, these women have to live with incontinence and strong odor that accompanies the condition and they are likely to face debilitating physical, psychological, social and economic consequences [16, 17].

The success rate of fistula repair is between 70-90 % if performed by an experienced surgeon, with appropriate nursing care and prevention of complications [18, 19]. Studies have shown that following successful fistula treatment, women could gain their reproductive health [20, 21]. Nevertheless, fistula repair services are overwhelmed by the increased numbers of new cases that occur each year [22]. Many countries with high prevalence of fistula lack adequate doctors with specialised training to treat fistulas. In Tanzania for example, in the year 2014, there were 225 doctors trained to perform fistula repair, and five health facilities offering fistula surgeries [23]. Despite the increase in number of doctors with specialised skills to treat fistula, and an increased number of fistula repairs conducted from 268 in 2010 to 500 in the year 2012, hospitals still have limited capacity in terms of infrastructure. Theatres and hostels where women and their relatives reside while awaiting for fistula repairs are still limited. Further, societies have inadequate information about fistula and its treatment [22] . This means many women would continue to live with fistulas for years and have to face the suffering associated with it.

Few studies on obstetric fistula have been conducted in Tanzania addressing social vulnerability issues [6, 24], experiences of birth care [25], religious coping of obstetric fistula [26] and women’s reintegration after fistula repair [20, 27]. This study describes how socio-cultural perceptions and practices of people on women living with fistulas affect women’s experience of living with it. The ‘three bodies’ framework was used as a lens to understand these experiences.

### The ‘three bodies’

Obstetric fistula is a fundamental bodily condition, which is perceived and experienced in particular cultural and social contexts. The ‘three bodies’ approach views the body as a physical and symbolic entity that is both naturally and culturally constituted [28]. The approach represents three levels of analysis. The *‘individual body’* is the biological body, which is acquired by birth and focuses on self-lived experiences including self- perception or image, feelings and sensations about the individual-self body. However, one’s self-understanding changes with the social context, within social relations. The *‘social body’* (the body as a symbol)*,* is a socially defined and culturally constructed body, the body that is needed in order to live within a particular society and cultural group. The social body influences and controls the physical functioning of individuals within a society and sustains particular views of society and of social relations. The *‘body politic’*, is about power and power relations. Body politic exerts control over all aspects of the individual body including behaviour in reproduction and sexuality, health and sickness, work and leisure and forms of deviance. The body politic shapes the body according to the needs of the society. Generally, the three bodies’ approach explains how bodies are understood and how different meanings are attached to the bodies, what is regarded as acceptable or unacceptable in the society, and the power and control exercised on the body.

## Method

### Study design and setting

This was a descriptive qualitative study where both individual interviews and focus group discussions (FGDs) were used to gather women experience of living with obstetric fistula. Combining both individual interviews and FGDs enhanced data richness and trustworthiness of the findings [29]. The study was conducted at the Comprehensive Community Based Rehabilitation in Tanzania (CCBRT) hospital. CCBRT is a private non-governmental organisation (NGO) in Dar es Salaam that serves as a major service delivery point for obstetric fistula repair in the Coast region. It also receives patients from the central and eastern part of the country. It performs about 400 vesico-vaginal fistula (VVF) and recto-vaginal fistula (RVF) surgeries each year. The hospital has a 21-bed fistula ward, and a hostel where fistula patients live while awaiting fistula repair, and has an active-case finding program that trace patients in rural areas and bring them to hospital for surgical treatment free of charge. The active-case finding program is achieved using mobile-phone based money transfer service to send cash to women with obstetric fistula for transportation costs to come to CCBRT.

### Selection of participants

Twenty-eight conveniently selected women lived with obstetric fistula participated in this study. The inclusion criteria included: 1) women with fistula admitted at the CCBRT hospital for surgical repair 2) ability to speak Kiswahili and 3) willing to participate in the study. Women, who met the inclusion criteria, were briefed about the purpose, method and principles of confidentiality, and thereafter a suitable time for interview was arranged. In total, 16 women (9 women before fistula repair and 7 after repair) were interviewed, whereas 12 participated in the FGDs.

### Interview procedure

#### Semi-structured interviews

Sixteen (16) semi-structured interviews [30] were conducted in Kiswahili, the language spoken by all participants and by the authors. Interviews were carried out at CCBRT hospital. Semi-structured interview guide was used which had topics and probing questions focusing on women experience of living with obstetric fistula. The guide was used flexibly to allow the interviewer to explore issues of relevance as they emerged [31]. In each interview, the participant was a major speaker and the researcher was mainly a guide and a facilitator. All participants spoke openly during interviews. The audio-recorded interviews lasted between 45 min to 2 h.

#### *Focus group discussion (FGDs*)

**Fig. 1:**
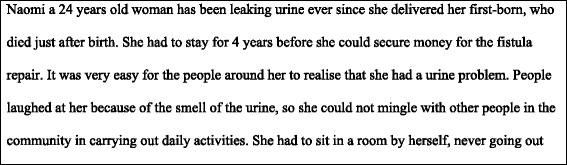
Hypothetical scenario presented before group discussions

Two FGDs each composed of six participants were moderated by the first author and attended by an observer and a note-taker. One discussion group comprised of women with obstetric fistula before they had fistula repair and the other with women after fistula repair. Participants were encouraged to participate actively in the discussion and were given equal opportunity to contribute. They were informed that there were no right or wrong answers. Discussions began by reading aloud a hypothetical scenario (Fig. 1), describing potential challenges faced by women living with fistula. The scenario was used as guide to stimulate the discussion. An audio recorder was used to record discussions and was supplemented by handwritten notes.

### Data analysis

A person fluent in Kiswahili transcribed the audio recorded interviews and FGDs. The Kiswahili transcripts were then translated into English and thereafter another person with good command of both Kiswahili and English crosschecked the accuracy of translations. Two researchers examined all transcripts for accuracy and completeness against the original notes before data was ready for coding. Gaps identified or clarifications needed were discussed and corrections made accordingly. The thematic analysis [32] approach guided analysis of the data. The procedure included familiarisation with the material, identification of the codes, searching for themes, revision of the themes and interpretation. All transcripts were carefully read sentence by sentence many times to become familiar with the content. Phrases and sentences related to the women’s socio-cultural experiences of living with obstetric fistula were coded in the margin of the transcripts. The codes that were similar or connected to each other were organised together to form themes. To strengthen content validity, multiple coding were considered, researchers scrutinised all codes [33] and finally agreed on formulation of themes.

### Ethical consideration

Ethical clearance was obtained from the Muhimbili University of Health and Allied Health Sciences (MUHAS) Research and Ethical Review Board, whereas the CCBRT hospital granted permission for data collection. Participants were fully informed about the purpose of the study and the use of audio recorder during interviews. It was made clear that information provided would be treated with strict confidentiality and would only be used for the research purposes. All names used are fictitious. All participants gave a written consent to participate in the study.

## Results

**Fig. 2:**
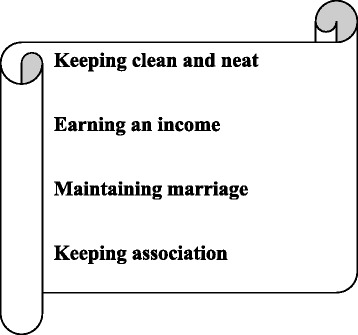
Women’s socio-cultural experience

Four themes illustrating socio-cultural experiences of women living with obstetric fistulas emerged from the analysis of women’s narratives on experiences of living with obstetric fistula. These were: keeping clean and neat, earning an income, maintaining marriage and keeping association (Fig. 2).

### Keeping clean and neat

Describing their experience with obstetric fistula, all women were deeply concerned about their inability to keep clean and neat. Women reported that they were always putting on ugly dresses, which reduced their value as women. It was not possible for them to wear their best clothes, and most of the time, they wrapped themselves up in old *‘*khangas’ or ‘vitenges’ (East African women fabric), in order to prevent the continuous dribbling of urine:

(…) you could have your best skirt and blouse but you cannot dress up, for fear of soaking, instead you just wrap yourself with pairs of old ‘khangas’ to protect yourself in case something goes wrong. Therefore, as a woman somehow there are certain deficiencies in your everyday life (Mwajuma, 35 years, 18 years with fistula).

The desire of women to keep clean and dress nicely was revealed during discussions:

(…) you cannot put on your best dresses, because if you dress well, after few minutes you ruin your dress with urine, consequently, you resort to putting on your ordinary clothes (…), many times, you just stay shabby (FGD- before fistula repair).

Other women thought that for a woman, failure to keep clean and dress nicely robbed their happiness, and self-esteem, and always envied other women who dress nicely and walk confidently:

(…) I am in sorrow (silent…), when you look at your friends, walking in their best dresses, while you cannot even dress nicely, it saddens me. (…) how could you dress well with urine leaking? (…) you just put on ugly dresses (Sara, 30 years, 3 months with fistula).

### Earning an income

It is widely known that financial independence brings about not only one’s freedom, but also happiness and confidence. Women living with fistula spoke of their inability to earn income and be independent as they used to be before developing fistula. Their experience of continuous leaking of urine, the smell, feelings of shame, fear and sorrow affected their ability to earn a living through farming, petty business, peddling or employment. The major obstacle was the inability to control the continuous leaking urine and odour:

(…) this problem has really slowed my progress (…) you cannot do any work, whether it is doing petty business or work in the office you cannot, because you will smell, your colleagues will smell urine, (…) frankly it is sad (Mercy, 35 years, 6 years with fistula).

(…) I am lost, I lost direction and money and my entire business has come to a standstill. (…) my life is in recession. I am not doing any business since I developed this problem (…) I cannot do anything, even if you put rags from ‘*khanga*’ as sanitary pads they hold on for just 3 to 5 minutes, so it is not possible to do business which require walking all day. How many sanitary changes could you make, and after all, those sanitary clothes will definitely smell and you will not be able to do business” (Jane, 26 years, 2 months with fistula).

(…) I was working as a house helper, I was earning an income, (…) for now I cannot work and earn an income (…) it hurts (Stella, 30 years, 6 years with fistula).

Financial reliance has always been an emotional struggle for women lived with obstetric fistula. Women who were earning money on their own before acquiring fistula, consistently described feelings of being hurt, insecure and reliant. Women expressed that they entirely depended on their husbands, parents and other people for financial support, because they were unable to sustain themselves:

(…) others are farming, I am not, I just stay at home, and my husband is the one who has to go to the farm and help me for everything (Clara, 25 years, 8 months with fistula).

Women who before they got fistula used to earn money and supported their families socially and financially, could no longer do so after developing fistula and this was a very painful experience:

(…) I had a job, I had a stable income, I could stand on my own (…), I used to send money to my parents, now I cannot. I now depend on other people to give me cash to buy even body lotion and soap (…). What hurts me most is the failure to help my mother, who is sick and there is no one to help her (crying…). I wish I could work as before and help my family (…) (Penny, lived with fistula for 19 years).

### Maintaining marriage

Procreation and attendance to domestic chores are among the ascribed roles of a married African woman. Living with fistula for many years, continuous leaking of urine and symptoms associated with it; affect women’s marital life because they cannot assume their culturally ascribed marital roles. Women in this study described their unpleasant feelings related to their inability to have intimate relationships with their husbands as a serious and painful experience:

(…) I cannot have sex with my husband. I could not do it at all; I have no hope if I will ever have sex. I do not have any feelings for it anymore and I am not sure if I will ever be able to have sex again. As a woman (silent…), this is a huge and serious problem (Pili, 20 years, 1 year with fistula).

(…) I used to sleep with my husband but now we sleep separately because of this problem and the smell. (…) since I got this problem, we have had no sexual relationship up to now. This is the most painful thing (Clara, 25 years, one year with fistula).

Women who had no children and lost their babies during birth that gave rise to fistula had strong desire to have children. However, many lost hope of bearing children in the future because they thought that women with fistula could not bear children, thus unable to achieve this socially and culturally valued goal:

(…) I feel bad, because I cannot live the way all my colleagues live (silent…), for example, I stopped having sex long time ago, perhaps if I was sexually active I could have had another child, but now that I don’t, how can I get a baby (crying…) (Sikitu, 43 years, 20 years with fistula).

Women were unable to carry out domestic chores. For example, they were not allowed to cook for the family, because they were considered dirty and unclean. Other women had to relocate to the homes of their parents and relatives:

I am now staying in my mother’s house. (…) I cannot work, (…) I could not wash his (husband) clothes nor do mine, I only clean my sanitary ‘rags’; my clothes are washed by my niece (Jane, 28 years, 2 months with fistula).

Following failure of women to attend to their marital obligations, the life of women with fistula is characterised by isolation, humiliation and rejection by the community. Women in this study were divorced, separated, asked to go back to their parents or abandoned by their husbands:

(…) I had a husband, but he left. He ran away because of urine (…). When he was leaving, he told me “I cannot tolerate and wait for you to heal” (Jenifer, 32 years, 19 years with fistula).

Another woman shared the experience of abandonment by her husband who moved away to start a new life:

(…) after I got this problem, my husband left home, he moved on completely to start a new life, and married another woman. Since he left he has never come back until now, and I am told he has a child (Penny, lived with fistula for 19 years).

Women lived with obstetric fistula reported that their husbands left them because of their inability to conceive and bear children. This made them feel hopeless, useless and valueless:

(…) I am useless and have no value. Had it not been for this problem, my husband would not have abandoned me. He left me because of leaking urine, and not being able to bear a child for him (Semeni, 28 years, and 12 years with fistula).

Jenifer felt hopeless:

(…) I feel hopeless and useless; I even lost the baby. It could have been better if my baby survived, because yes, you have a problem, but you still have your child; it would be much easier.

For other women the trauma of losing children was experienced as grief:

(…) I often asked myself, how would my life be without a child? (Maua, 24 years old, lived with fistula for 5 years).

Despite the fact that many women spoke of experiences of lacking support from their husbands, few reported receiving support from them:

Yes, (…) my husband supported me. He provided me with cash so that I could seek fistula treatment (Clara, 25 years, one year with fistula).

### Keeping association

Continuous leaking of urine and the smell associated with it pushed women away from attending to spiritual observances or community gatherings as part of their social responsibilities. Women could not go to parties, church or to mosque for prayers, visit friends and families nor attend funeral ceremonies that are culturally important and essential activities in maintaining social networks:

(…) You cannot go to the mosque for prayers or travel. (…) with this problem, it is difficult to socialize with friends, or entertain guests freely and peacefully. It is better to stay at home, because you know your schedule, like now is time to wash clothes etc. but it is very difficult if you are far away from home (FGD- before fistula repair).

(…) You cannot go to church, you cannot go anywhere because of the smell and the leaking of urine. You feel ashamed when people look at you. Even when there is a funeral in your family, you only go to the graveyard; thereafter you go back and sleep at home, whereas others sleep in the deceased house as per tradition. (…) how could you sleep in other peoples’ houses when you are leaking urine? (FGD- after fistula repair).

Sara, emotionally spoke about this issue, she said:

(…) I feel as if the inside of me is rotten. (…) I cannot sit and chat with colleagues, because all the time I feel wet and smelly (Sara, 30 years, 3 months with fistula).

## Discussion

### Living with obstetric fistula and the ‘*Individual body’*

Because of women’s inability to restrain urine and or faeces, and the resulting smell, genital sores, discomforts, feeling of shame and stigma; women were not welcome to participated in socio-economic activities nor contribute to the family earnings and development as expected. Women were unable to work, or work to their capacity because fistula limited their chances to access jobs. Therefore, women could not earn an income through farming, business, hawker or employment. Studies have reported that women who were employed lost their jobs because of the condition [17]. In general, women experienced severe reduction in their source of independent income, and increased their dependence on others [34]. Women in this study had bad feelings about their inability to earn a living and failure to support their family financially, as their financial assistance to their families and relatives was important. It is likely that due to failure of these women to support their family and relatives, resulting in the whole family to revolve into the poverty circle, especially if the woman was the family’s breadwinner. Overall, being economically independent is directly linked to the sense of pride and respect.

Following constant leaking of urine and smell, it is very difficult for women living with obstetric fistula to keep clean and neat. It was hard for them to get cash that would allow them to buy scented soap and lotion to alleviate smell. Studies [35] have reported that women who lived with obstetric fistula used scented powders and perfumes to cover for the offensive odour. However, many women with obstetric fistula in this study and others [9, 20] were from remote rural areas where water supply is frequently unavailable, lack basic education and have no stable income.

Women’s responses during interviews and discussions regarding their experience of living with a fistula were how would *‘other people’* think, see, and speak about them with respect to the leaking of urine*?* At large, women experience reflected the societal perceptions about an ideal woman and their knowledge about obstetric fistula. However, peoples’ perceptions on women affected by obstetric fistula may be linked with their poor knowledge and misconceptions about causes, treatment and prospects after fistula treatment [36, 37]. Many people do not know that after fistula treatment women could regain their reproductive abilities, conceive and deliver live healthier babies [20, 21]. Women affected by obstetric fistula in this study were perceived as abnormal due to the leaking of urine or faeces and the smell. These experience can also be explained by “the looking glass self” concept [38], that women living with obstetric fistula shape their self-concept basing on the reflections of the response and evaluations of other people around them, and the understanding and perceptions of obstetric fistula in the society.

### Living with obstetric fistula and the ‘*social body’*

A woman in most of African cultures is expected to assume ascribed marital and social responsibilities. Continuous leakage of urine and or faeces, the foul smell and genital sores make the woman unable to assume her socio-cultural expected roles. A healthy sexual life is the source of children and family bonding, lack of it contributes to women loss of self-esteem about their womanhood [39]. Women in Tanzania, as in many Sub Saharan African societies acquire value from their husbands and society through marriage. They gain sense of self-esteem through their ability to bear children and fulfil their roles as women and wives [40, 41]. Full womanhood is attained through being a mother and there is no worse misfortune for a woman than being childless. A barren woman is regarded as incomplete [42]. Therefore, having children is the most fundamental value for women and men [42, 43], as it is commonly perceived as the main purpose of ones being. Living with uncontrollable leaking of urine and symptoms associated with it for many years prevent women from having sexual intercourse with their husbands and bearing children. Many women affected by obstetric fistula lost their babies during the birthing process [41]. Therefore, the need for having a baby is obvious [27]. Nearly all study participants had strong negative feelings about their inability to have sex with their husbands because this denied them intimacy and bonding expected in marriage and desire to have children.

In the eyes of the society ‘*the social body’*, women who had no children were considered as humans of lesser value and may at times become targets of many rude insults and impolite treatment. Eleven of 28 women in this study were divorced or abandoned by their husbands, findings that are consistent with those found by others [17, 41]. As indicated in other studies [35] husbands of women living with fistula divorced their wives because they were disgusted with the condition and their wives inability to have children. Generally, the husband as an individual does not make the decision to divorce his wife, rather is a family matter. It is common for the husband’s family members and relatives to ask the husband to divorce his wife and marry another woman who would bear children [44].

The respect, honour, and sense of pride associated with having children [24, 40] may explain why women who had children before developing fistula had a relatively more positive experience of living with fistula than their counterparts who did not have children [41]. Studies [24, 45] have reported that women who had children before fistula occurrences had their husbands beside them for support. Husbands commonly allowed their wives to live in the family compound, cook and assist with children rearing even if they would opt to have relationships with other women. Women consistently reported that it would have been better if they had children. It is likely that these women compared their experience to those who had children before they had fistula. Many women lived with fistula who managed to sustain their marriages however, they reported to have had no intimate relationships with their husbands [44].

### Obstetric fistula and the ‘*body politic’*

Obstetric fistula occurs when labour is allowed to progress for a period lasting from several days to a week without quick intervention, usually a caesarean section [46, 47]. In Tanzania many women live far from where adequate health facilities are located [25]. Although primary health facilities are within 10 kilometres reach, these facilities however do not provide comprehensive emergency obstetric care (CEmOC) [48]. CEmOC health facilities are those capable of providing basic emergency obstetric care (BEmOC) and also performing caesarean section and blood transfusion. Studies have shown that getting to the health facility, does not assure women access to quality birth care [25]. Health care facilities especially those in rural areas are often understaffed with inadequate utilities, equipment, supplies and medication [49], and commonly lack skilled personnel [50]. In spite of the national health policy emphasis on equity in availability and access to health care, this has not yet been realised. Many women still give birth at home [50]. Only 50 % of women in Tanzania give birth in the health facilities and about 51 % of these receives skilled birth care [1]. Increasing the proportion of births with skilled attendance is advocated as one of a key factor in reducing maternal and perinatal mortality and morbidity [51].

Maternal mortality and morbidity in Tanzania is still unacceptably high [1]. Experiences from countries with low rates of mortality and morbidity reveals that access to good quality obstetric care is vital in reducing maternal mortality including obstetric fistula [51]. To influence access and quality of obstetric care for all individuals in reproductive age group in Tanzania, the Government must ensure availability of skilled personnel and an enabling environment for them to work effectively. Motivated skilled staff [52] and solid health systems are associated with provision of quality services [3]. The total budget allocation for health need to be increased to at least 15 % as suggested in the Abuja Declaration in 2000 as the minimum budget for health [53] to improve quality of obstetric care. Solid health systems would help in early diagnosis and management of obstetric complications and thus preventing obstetric fistula. Further, the Government has to ensure that women living with obstetric fistula get treatment by ensuring that many Government health facilities engage actively in performing fistula surgeries. Rehabilitation mechanisms for women affected by obstetric fistula put in place to increase women’s access to fistula treatment, improve their sense of worthiness, and maintain their dignity as women and wives in the eyes of people in the society.

This study demonstrates that socio-cultural context largely influenced women experience of living with obstetric fistula. Drawing from the health promotion perspective, an individual’s wellbeing is best considered within the context of the family, and the family within the context of its community [54]. Women experience of social exclusion and the feelings of worthlessness in this study are mainly a response on how people in the society perceived a woman as “normal or natural” basing on the socio-cultural understanding that in turn influences their actions towards women living with fistula.

### Limitation of the study

This study was limited to 24 women affected by obstetric fistula. Since women were conveniently recruited from CCBRT, a specialised hospital for fistula repair, it is likely that women who managed to seek care were those who received support from their relatives and communities, whereas those who could not come to the hospital had more severe negative experiences of living with fistula. Nevertheless, this study did not aim at transferability or generalizability of findings, but rather to understanding and shed light on how women experience living with fistula in Tanzania.

## Conclusion

This study unveils the day-to-day experience of social discrimination and loss of independent income of women living with fistula. Women who live with fistula are not able to assume ascribed socio-cultural responsibilities. They are a burden because they cannot contribute to the family earnings, cannot satisfy their husband’s sexually, nor bear children. Therefore, in the eyes of the society they are of less value due to failure to carry out social and marital roles. Lack of society awareness about fistula and availability of treatment and issues related to fertility after fistula repair have contributed to the negative perceptions by societies about the affected women and in turn women’s experience of social discrimination. Education programs through various sources to communities will help people understand about fistula including the nature of the condition and its cause so that women living with fistula are welcomed and receive fistula treatment and necessary social support.
